# Key factors in developing effective digital health promotion tools for cancer prevention and health behavior change in adolescence through a multi-country survey

**DOI:** 10.1186/s12889-026-26412-6

**Published:** 2026-02-21

**Authors:** Vassilis Kilintzis, Haridimos Kondylakis, Aristeidis Petrakis, Kleio Koutra, Katerina Micheli, Magdalini Pelekidou, Chariklia Tziraki, Emmanouil Tsiknakis, Severin Haug, Nikolaos Boumparis, Nikolai Kiselev, Laura Maria Del Campo, Eunate Arana Arri, Polonca Serrano, Teresa de Pablo Pardo, Stefanie Verduyn, Gloria Cea, Alba Gallego, Maria Krini, Andreas Triantafyllidis

**Affiliations:** 1 Hellenic Mediterranean University, Heraklion, Greece; 2 University Zurich, Zurich, Switzerland; 3 Federazione Italiana Delle Associazioni Di Volontariato In Oncologia (FAVO), Rome, Italy; 4 BioCruces Health Research Institute, Barakaldo, Spain; 5 Alma Mater Europaea University, Maribor, Slovenia; 6 Fundación Para El Fomento de La Investigación Sanitaria y Biomédica de La Comunitat Valenciana, Valencia, Spain; 7 Vlaams Instituut Gezond Leven, Brussels, Belgium; 8 BRIDG OÜ, Tallinn, Estonia; 9 Cyprus Association of Cancer Patients and Friends (PASYFAK), Nicosia, Cyprus; 10 Centre for Research and Technology Hellas, Thessaloniki, Greece

**Keywords:** Digital Health Promotion, Multi-country survey, Health Behavior, Cancer Prevention, EHealth, Co-Creation Methods

## Abstract

**Objectives:**

Primary cancer prevention through behavior change in adolescence, a crucial period for shaping lifelong health habits, presents a major public health challenge across Europe. Addressing this, the SUNRISE project aims to tackle the challenge of primary cancer prevention in adolescents by developing and implementing an innovative, digitally-enhanced life-skills program tailored to diverse socio-economic, cultural, and environmental backgrounds by incorporating various Digital Health Promotion (DHP) tools to foster sustainable health behavior change in adolescents. The present study aims to identify key requirements and features that should be considered while developing effective DHP tools, based on a multi-stakeholder survey conducted across seven European countries.

**Methods:**

The survey was conducted on 505 stakeholders (students, parents, and educators), from seven European countries to assess a set of key features for effective DHP tools for their importance on a five-point likert scale.

**Results:**

Our findings revealed that seven of the proposed DHP tools’ features were identified as important considering all the stakeholder groups, while significant differences in the importance of certain features across different stakeholder groups and countries were identified. Students, as primary users, demonstrated distinct preferences, which often diverged from educators and parents, suggesting that stakeholders hold distinct priorities driven by their roles and contextual backgrounds. Additionally, country-level variations were notable; for example, Swiss participants rated the proposed features, in general, as of lower importance than the Spanish respondents.

**Conclusions:**

These insights emphasize the necessity of developing adaptable and context-sensitive DHP tools that reflect the diverse needs and preferences of adolescents across Europe. The large-scale implementation and evaluation of this program will provide valuable data for shaping future digital health interventions aimed at cancer prevention in youth.

**Supplementary Information:**

The online version contains supplementary material available at 10.1186/s12889-026-26412-6.

## Background

Primary prevention of cancer through behavior change during adolescence—a developmental stage when many health-related behaviors are established—presents a critical health and societal challenge across Europe [1] and it is recognized as a priority prevention target by major public health bodies. Behavior change during adolescence requires targeting key modifiable risk factors such as obesity, tobacco use, harmful sun exposure, and low HPV vaccination uptake, all of which are strongly associated with future cancer-related morbidity and mortality [2]. Because these risk behaviors typically emerge and solidify before age 18, and adolescence represents a critical developmental window of “cumulative risk” for later-onset cancers, early intervention is essential for preventing cancers associated with these exposures across the life course [3].The present study focuses on adolescents aged 12–19 years, a range that captures early, middle, and late adolescence—stages marked by increasing autonomy, greater exposure to risk environments, and the formation of health decision-making patterns that often persist into adulthood.

Recognizing this need, SUNRISE EU-funded project [4] seeks to co-create, implement, and evaluate an innovative, digitally enhanced life-skills program designed for primary cancer prevention, with a strong focus on promoting sustainable health behavior change among adolescents. Specifically, SUNRISE focuses on preventing tobacco use and exposure to second-hand smoke, promoting healthier dietary habits and regular physical activity to support healthy body weight. In addition, SUNRISE seeks to strengthen sun-safe practices to minimize ultraviolet (UV) radiation exposure and to encourage timely uptake of HPV vaccination where relevant. Recognizing that psychosocial factors also shape long-term health trajectories, the program incorporates activities that foster stress management, social connection, and adolescents’ confidence in making health-promoting decisions. Together, these targeted behaviors form the foundation of sustainable cancer-preventive habits during a pivotal developmental period. Additionally, SUNRISE is customized to consider the socioeconomic, cultural, and environmental diversities that characterize European youth and to achieve its objectives, it integrates a validated, evidence-based digital solution for cancer prevention with cutting-edge intervention strategies, including peer-driven social media campaigns, advertising literacy training, educational games, and interactive platforms featuring social robots. This multi-faceted approach aims to enhance cancer prevention efforts among adolescents across Europe.

Digital platforms have the potential to disseminate information rapidly to a large number of people [5] and Digital Health Promotion (DHP) tools are essential in fostering healthy behaviors and empowering individuals to take proactive roles in managing their well-being by modifying behaviors that influence preventable disease risk factors [6, 7]. According to several comprehensive literature reviews [8–11], a variety of technological platforms are employed in DHP tools, including computer- and web-based programs, smartphone apps, and telemonitoring devices such as sensors. The effective development of DHP tools demands careful consideration of multiple factors to optimize usability, user engagement, and overall impact. Biomedical Informatics textbooks [12] and country-specific identification of digital health trends and challenges [13] are available and may act as a starting point for developing DHP tools. At the same time, adolescents report important challenges, such as complex or burdensome navigation, lack of contextual personalization, privacy and confidentiality concerns—particularly for sensitive topics—digital literacy gaps, and exposure to misinformation when content is not evidence-based. Youth also note that limited human interaction in digital interventions may reduce trust and emotional support, and that high notification frequency or time-consuming data entry can undermine continued engagement [14]. These findings underscore that although digital tools offer unique opportunities for scalable, flexible, and youth-centered cancer prevention, their effectiveness depends on thoughtful co-design with adolescents, careful attention to interface and content design, and safeguards that ensure privacy, accuracy, and sustained user engagement. Despite growing research on digital health interventions for adolescents, the current bibliography lacks evidence from multi-country, multi-stakeholder studies examining the requirements for digital health tools toward cancer prevention in adolescence.

The present study aims to expand current knowledge regarding the essential application features that should be considered while developing effective DHP tools based on a survey conducted across seven European countries involving key stakeholder groups, namely secondary school students, educators, and parents.

## Methods

The SUNRISE project and its components are being developed through a co-creation process, using a "schools-as-living-labs" model [15] that engages diverse societal stakeholders. These include educators, adolescents, parents, public health professionals, and policymakers. The program is set for large-scale implementation and evaluation, spanning 154 schools and reaching over 7,500 students in both urban and rural regions across several European countries. Special attention is given to the inclusion of socially disadvantaged groups, such as migrants and ethnic minorities, to ensure the program’s equity and inclusivity. In addition to assessing the efficacy of its methods for promoting long-term health behavior changes, SUNRISE will evaluate strategies for widespread adoption and sustainability across multiple countries.

The primary aim of this survey was to identify the most critical requirements and features necessary to develop effective DHP tools targeting adolescents aged 12 to 19 years. Specifically, the study examined features that support health behavior change relevant to primary cancer prevention during adolescence, a critical developmental period when many risk-related behaviors emerge and may become lifelong habits [16, 17]. By identifying and prioritizing these essential features, the survey provides evidence-based guidance for the co-creation and development of DHP tools designed to resonate with adolescent users while also addressing the needs of other key stakeholders.

### Survey design

The survey incorporates elements identified in an extensive literature review [18] presenting the aspects related to preferred content of digital health promotion platforms after evaluation of 16 studies in the field [18]. These aspects underwent a review by a multidisciplinary team, including technical, social science, and health experts, that adapted them and contextualized them to align with the aims of SUNRISE. The review process ensures the alignment of the proposed aspects (proposed DHP tools’ features) to the aim of identifying the key factors for developing effective and engaging DHP tools for cancer prevention and health behavior change in adolescence.

During the implementation of the survey, the proposed features (presented in detail in Table 1) were rated on a five-point likert scale by the survey participants representing three primary stakeholder groups: students, educators, and parents. These groups were selected to capture diverse perspectives on health-promoting features, ensuring that the needs and preferences of end-users (students) and influential adult stakeholders (educators and parents) were represented.

**Table 1:** The list of the proposed DHP tools’ features that were rated by the survey participants

ID	Proposed feature for effective DHP tools	ID	Proposed feature for effective DHP tools
Ft1	Tailored/personalized content (incl. for age, gender or where you live)	Ft 15	Trackers for diet, exercise (incl. tracking progress and awards for completion)
Ft 2	Trusted content presenting source of information (e.g., health professional such as a dietitian, endorsed by a university or government organization)	Ft 16	Engaging content (e.g., videos, quizzes)
Ft 3	Information on multiple health behaviors (e.g., diet, physical activity, sedentary time, BMI)	Ft 17	Immersive content (e.g., games, interactive components)
Ft 4	Specific and relevant (‘themed’), rather than general	Ft 18	Achievable and monitored goal setting with feedback (via app or website)
Ft 5	Positive/affirming content, rather than negative content (e.g., avoid terminology like child obesity and weight management) that elicit negative reactions	Ft 19	Informative content (e.g., facts, health benefits, nutritional information)
Ft 6	Practical ways to improve behaviors (‘how to’ guidance)	Ft 20	Videos (e.g., online demonstrations)
Ft 7	Budget-friendly information (i.e., suggestions that do not have a high economic impact)	Ft 21	Resources related to local area (e.g., open sport places, farmers markets, message board for events)
Ft 8	Regularly updated content	Ft 22	Customizable, based on personal user accounts
Ft 9	Content focusing on multiple topics	Ft 23	Reminders/notifications/messages, including via email or SMS
Ft 10	Features relevant for/to involve the whole family (e.g., games, area or activities for children, cooking with children, sections for parents)	Ft 24	App delivered for free
Ft 11	Ability to post questions to health professionals (e.g., via a live chat interface, contact box, video chat) or regular contact with health professionals	Ft 25	In-app search function
Ft 12	Ability to connect/interact with other users, including via a discussion forum, social media, Facebook chat	Ft 26	Offline access to content/activities
Ft 13	Practical shopping tools: shopping lists, barcode scanners, ingredient calculators	Ft 27	Accessible via smartphone
Ft 14	Recipes (budget-friendly, child-friendly, quick, healthy, linked to seasonal produce)	Ft 28	Accessible via laptop/PC

### Participants and recruitment

The survey was conducted across seven European countries: Cyprus, Greece, Italy, Slovenia, Spain, Switzerland, and Belgium. This cross-national approach provides insights into feature preferences across different cultural and regional contexts, enhancing the DHP tool's potential adaptability and relevance across the European Union. The research teams in each of the collaborating sites were asked to recruit (without strict proportions requirement) ~ 70 participants for responding in the anonymous survey using their local network. The inclusion criteria were limited to adolescents, parents of adolescents and high school educators considering, also, the inclusion of participants with diverse ethnic backgrounds. The collective target was to recruit 500 participants.

### Data collection

The survey was executed via a self-hosted electronic survey tool [19] after Ethical approval (number: 11068, 11/04/2024) was obtained from the Committee of Research and Ethics of the Hellenic Mediterranean University. Before the official launch, the survey’s comprehension and functionality were pretested in each participating country. To this end, at least two individuals from each country reviewed the survey by navigating through it and providing feedback to the research team. These responses were systematically evaluated, and necessary modifications were incorporated to enhance clarity and usability. The survey was completely anonymous, without storing any identifiable information of the participant or his/her device (IP address, date of submission etc.), and, thus, disabling any link between response data and participant. To achieve distinction among responses from different countries, a unique link per country was shared, and the survey was presented in either the local language or English, based on the respondent’s preference. The translation process followed a gold-standard approach to ensure linguistic and conceptual equivalence across languages. Initially, a bilingual researcher translated the questionnaire into the target language. Subsequently, an independent person with equivalent qualifications performed a back-translation into the original language. Any discrepancies between the original and back-translated versions were discussed and resolved collaboratively to maintain accuracy and consistency. Based on the anonymous structure of the survey, only completed surveys (i.e., the participant navigated through all the questions and pressed the final submit button) were allowed to be included in the analysis.

Although the respondents in each country were approached differently, in general, the research team employed a convenience sampling method for participant recruitment. The invitation to complete the survey was disseminated through the research network of the participating institute and there were no incentives for participation.

Each of the 28 features was evaluated on a five-point Likert scale, where participants rated each feature’s importance from 1 (Not important at all) to 5 (Very important). This method allowed us to gauge both the high-priority features essential for user engagement and the lower-priority features that may be less impactful in the final tool design. Surveys were completed, without supervision, to encourage honest and thoughtful responses from participants. For adolescent participants, parental consent was required, adhering to ethical standards in research involving minors.

### Data analysis

Our primary analytical objective was to systematically identify the most critical features as rated by stakeholders. To accomplish this, we categorized survey responses that selected "5. Very important" as reflecting high importance and grouped those marked as "1. Not important at all" or "2. Not that important" as indicative of low importance. Following this we calculated the proportion of high importance ratings (HIGH) (computed as the ratio of responses marked as "5" to the total number of responses for the specific feature) and the proportion of low importance ratings (LOW) (computed as the proportion of responses marked as "1" or "2").

A feature was, then, classified as "possibly important" if it met two conditions simultaneously: (a) it presented a HIGH percentage greater than the average HIGH across all features, and (b) it presented a LOW percentage lower than the average LOW across all features. This dual criterion was adopted to ensure the identification of features widely recognized as valuable by stakeholders. Specifically, HIGH above the variable mean suggests broad consensus regarding a feature’s importance, as well as a LOW below the mean implies limited disagreement. Conversely, features exhibiting high percentages in both HIGH and LOW categories indicate divided opinions, making their inclusion less clear-cut. Thus, our approach emphasizes selecting features that receive consistent, unequivocal support.

To validate the results of our selection of “possibly important” features, statistical tests were conducted to ascertain if the identified as "possibly important" features significantly deviated from the rest. To achieve this, we implemented a proportion test to assess the null hypothesis that the tested feature’s proportion(percentage) does not statistically differ from the overall mean percentage. We reject a hypothesis at a 5% significance level. Only features that reject both null hypotheses—demonstrating statistically significant above-average HIGH scores and below-average LOW scores—are conclusively considered important for integration into DHP tools.

**Fig. 1:**
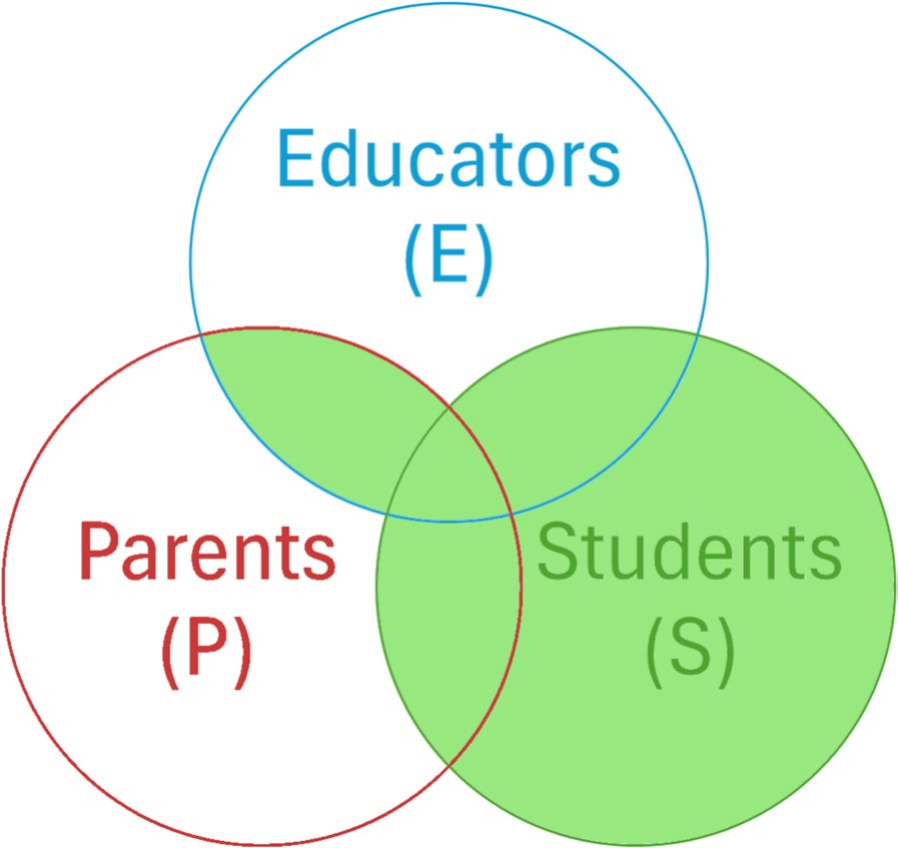
Final selection of features. Each circle represents the features that are important to the corresponding stakeholder group. The final selection is represented by the light green section (i.e., S ∪ (E ∩ P)))

In case there is a stakeholder group that is outnumbering significantly the others, there is an inherent bias that could influence the results in this co-creation process. To address this, we test the preferences for the statistically significant features within each stakeholder subgroup individually. For each group—students, educators, and parents—we repeated the “high” and "low" importance method, treating the average values for each subgroup as thresholds. These subgroup analyses allowed us to cross-validate which of the features were consistently important across groups. The final selection of features prioritizes the preference of the students by including all of their preferred features, while it includes, as well, the features that are important to both educators and parents (Fig. 1). This could ensure that the application remains student-centered while also reflecting broader stakeholder input. Finally, we compare the results based on all answers and the results obtained based on Fig. 1.

In addition to stakeholder-specific comparisons, we sought to examine whether the importance attributed to each feature varied significantly across countries. Firstly, we plot the High and Low preferences for each country in two separate figures, then to test whether perceived feature importance differed between countries, we compare the distribution of Likert ratings for every feature across all possible country pairs. Because the data are ordinal and may violate normality, we used the Wilcoxon rank-sum test (Mann–Whitney U) for each pairwise comparison.

For a given feature *f* and countries *A* and *B*, the null and alternative hypotheses were.

$$\mathrm H\_0\;:\;\mathrm R\_\left(\mathrm f,\mathrm A\right)\;=\;\mathrm R\_\left(\mathrm f,\mathrm B\right)\;\mathrm{vs}\;\mathrm H\_1\;:\;\mathrm R\_(\mathrm f,\mathrm A)\;\neq\mathrm R\_(\mathrm f,\mathrm B)$$

where R_f,A and R_f,B denote the distributions of ratings in the two countries. In other words, we asked whether a randomly chosen respondent from country *A* is equally likely to give any rating as a randomly chosen respondent from country *B*. We adjusted the resulting *p*-values with the Bonferroni correction to control the family-wise error rate and declared differences significant at α = 0.05.

For every significant comparison, we also recorded the Hodges–Lehmann location shift (the Wilcoxon estimate of the median difference). A positive shift indicates that ratings in country A tend to be higher than those in country B; a negative shift indicates the reverse; a value of zero denotes a tie. This direction indicator serves two purposes: it tells us which country assigns greater or lesser importance to a given feature, information that the p-value alone cannot provide, and checks the consistency with the figures.

All statistical analyses were conducted using R Statistical Software (v4.3.2; R Core Team 2021).

## Results

### Descriptive statistics

**Fig. 2:**
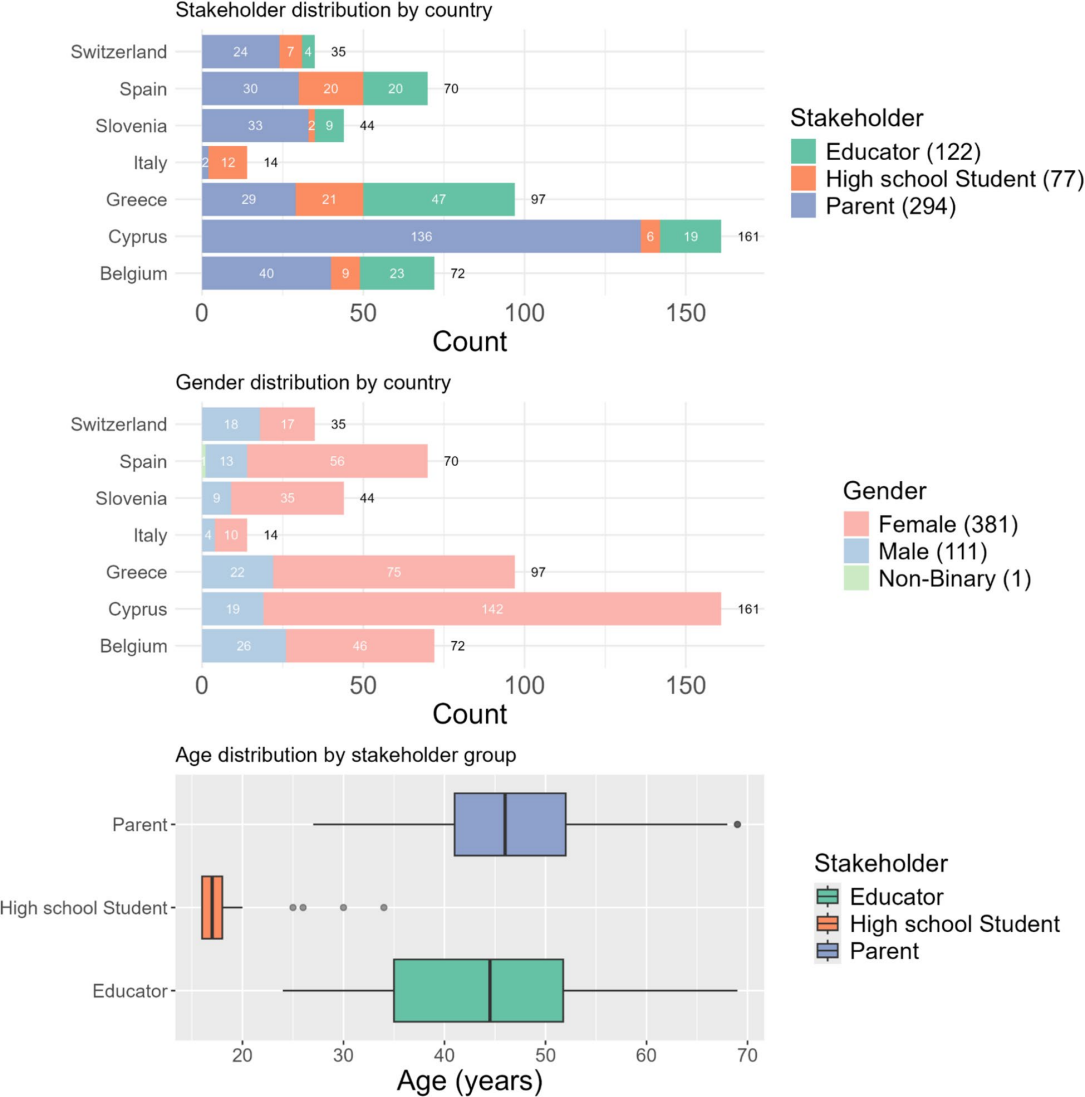
Key demographic characteristics of survey participants, including stakeholder type, gender, country and age distributions across respondent groups

A total of 493 completed surveys were collected, each reflecting at least 80% of the proposed features rated. The stacked-bar overview (Fig. 2) shows the participation pattern across the seven countries: Cyprus contributed 165 respondents, followed by Greece (97), Belgium (72), Spain (70), Slovenia (44), Switzerland (35), and Italy (14). Parents dominate the sample, especially in Cyprus and Belgium, whereas high-school students form the largest share in Italy (12 of 14) and constitute a sizable minority in Greece and Spain; educators are relatively well represented in Spain and Cyprus. Across every country, the gender split is skewed toward women: females account for 381 of the 493 respondents, with 111 males and one participant identifying as non-binary. The median age of the participating educators was 44.5 years, for high-school students 17 years, and for parents 46 years (Fig. 2). Finally, regarding the “born-in-country” status, 34 participants declared that they were not born in the country they currently live in. The presented participation pattern supports that the survey responses were gathered from a diverse set of participants regarding gender and born-in-country status, along with the planned diversity per stakeholder group and country.

Table 2 summarizes each feature's evaluation, reporting, the number of valid responses per feature, the percentage of high importance ratings (HIGH), and the percentage of low importance ratings (LOW), along with, the average HIGH and LOW values across all features. Highlighted by a preceding asterisk are the features with above‑average HIGH and below‑average LOW percentages, indicating the features identified as “possibly important” in our methodology. As presented in Table 2, the majority of participants rated most features as either "4" or "5," indicating a general consensus that these features are considered valuable. This skewed distribution suggests a high rating of feature importance across groups, which was expected due to the origin of the proposed features. Overall, the table shows that only a subset of the features (specifically, 13 out of the 28) attracted consistently high endorsement (i.e., above average HIGH, below average LOW).

**Table 2:** Distribution of Ratings per feature and corresponding LOW and HIGH rating proportions

		Rating, 1: Not important at all, 2: Not that important, 3: Not sure, 4: Important, 5: Very important					Average(HIGH) = 36.39% Average(LOW) = 8.11%	
Feature	Total Responses	[1]	[2]	[3]	[4]	[5]	HIGH [5] %	LOW [1, 2] %
Ft1*	478	9	15	54	209	191	39.96%	5.02%
Ft2*	486	4	13	32	178	259	53.29%	3.50%
Ft3*	487	3	9	23	186	266	54.62%	2.46%
Ft4	484	5	17	69	230	163	33.68%	4.55%
Ft5	489	13	38	75	155	208	42.54%	10.43%
Ft6*	489	3	4	17	182	283	57.88%	1.43%
Ft7*	489	6	14	52	190	227	46.43%	4.08%
Ft8*	488	2	17	23	200	246	50.41%	3.89%
Ft9	486	5	47	108	219	107	22.02%	10.70%
Ft10*	490	5	22	51	211	201	41.02%	5.51%
Ft11*	490	4	29	46	191	220	44.90%	6.73%
Ft12	489	19	77	138	175	80	16.36%	19.63%
Ft13	481	15	65	107	206	88	18.30%	16.67%
Ft14*	489	2	19	31	233	204	41.72%	4.30%
Ft15	483	10	31	81	228	133	27.52%	8.49%
Ft16	488	8	41	67	233	139	28.48%	10.04%
Ft17	488	6	39	90	218	135	27.66%	9.02%
Ft18	484	8	21	72	249	134	27.69%	5.99%
Ft19	490	4	19	34	257	176	35.92%	4.69%
Ft20	482	10	39	80	244	109	22.62%	10.16%
Ft21*	485	3	30	49	219	184	37.94%	6.80%
Ft22	482	15	45	131	196	95	19.71%	12.45%
Ft23	484	14	84	107	196	83	17.15%	20.25%
Ft24*	488	5	17	31	129	306	62.70%	4.51%
Ft25*	485	9	18	61	212	185	38.14%	5.67%
Ft26	483	5	41	63	207	167	34.57%	9.50%
Ft27*	483	5	13	42	164	259	53.63%	3.73%
Ft28	485	13	48	56	220	148	30.52%	12.58%

^*^Feature is possibly important: It presents a HIGH percentage greater than the average HIGH across all features, and, at the same time, it has a LOW percentage lower than the average LOW across all features

### Feature selection

**Fig. 3:**
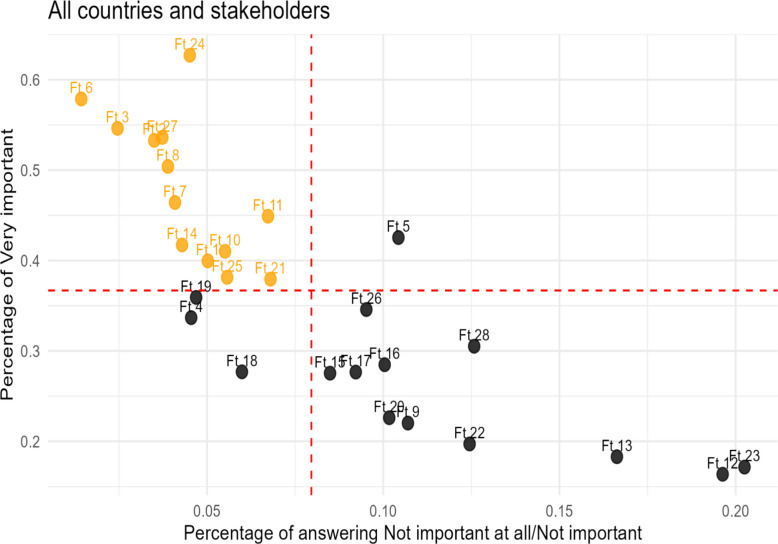
Overall preferences over the Features

In Fig. 3, we visually represent our methodological approach for identifying the important features. Each feature is positioned in the scatterplot using LOW on the X-axis and HIGH on the Y-axis. Lines representing the overall means of HIGH and LOW, across all features, intersect to form quadrants. Features located in the top-left quadrant—those scoring above-average in HIGH and below-average in LOW— are considered as “possibly important” features.

The 13 features situated in the top‑left quadrant of the figure, signaling their favorable combination of broad endorsement and limited disagreement, constitute the most compelling consensus candidates for important features of DHP tools.

Subsequently, Table 3 complements Fig. 3 by providing the statistical evidence on whether the distance of a feature from axis-X and axis-Y is statistically significant. In the table, for each of the 13 “possibly important” features, the p‑values from one‑sided proportion tests that compare each feature’s HIGH and LOW proportion with the overall means of HIGH and LOW, respectively, are presented. The nine features with a p-value below 0.05 are considered endorsed by participating stakeholders for inclusion in Digital Health Promotion (DHP) tools.

**Table 3:** p-values of the proportion test of Possibly Important Features

Feature	HIGH	LOW	Significant difference from the mean in both axes
	(*p*-value)	(*p*-value)	
Ft 1	0.066	0.005	No
Ft 2*	< 0.001	< 0.001	Yes
Ft 3*	< 0.001	< 0.001	Yes
Ft 6*	< 0.001	< 0.001	Yes
Ft 7*	< 0.001	< 0.007	Yes
Ft 8*	< 0.001	< 0.004	Yes
Ft 10*	0.024	0.020	Yes
Ft 11	< 0.001	0.149	No
Ft 14*	0.012	0.001	Yes
Ft 21	0.284	0.149	No
Ft 24*	< 0.001	0.002	Yes
Ft 25	0.253	0.02	No
Ft 27*	< 0.001	< 0.002	Yes

^*^Significant difference from the mean in both axes

Building on the overall feature‑selection results, the subgroup analysis offers a more nuanced understanding of stakeholder priorities. Table 4 reports the LOW, HIGH values along with the corresponding p-values of the one‑sided proportion tests for the features that are characterized as significant from at least one stakeholder group, by applying the same dual criterion, significantly (*p* < 0.05) above‑average HIGH and significantly below‑average LOW (i.e., any region of Fig. 1, (P), (E) or (S)). As Table 4 exhibits, seven features (Ft2, 3, 6, 7, 8, 24, 27) are significant for either the students, or, both parents and educators, thus, they belong to the green area of Fig. 1, i.e., S ∪ (E ∩ P). In detail, two universally supported features (Ft 6 and Ft 8) were identified, being (E ∩ P). In detail, two universally supported features (Ft 6 and Ft 8) were identified, being significant for all stakeholder groups. Moreover, parents and educators converge on three additional priorities—Ft2, Ft3, and Ft 7. In contrast, students align with parents only on Ft24 and with educators only on Ft 27. Lastly Ft1 and Ft14 were significant for parents but for no-one else. significant for all stakeholder groups. Moreover, parents and educators converge on three additional priorities—Ft2, Ft3, and Ft 7. In contrast, students align with parents only on Ft24 and with educators only on Ft 27. Lastly Ft1 and Ft14 were significant for parents but for no-one else. Aggregating these findings (Table 3 and Table 4), two features (Ft 6 and Ft 8) remain unequivocal priorities across every analysis: they are rated “very important” and seldom “unimportant” both on partitioned data and the whole dataset. Finally, two items that appeared in Table 3 (Ft10, Ft14) lose significance once the data are partitioned.

**Table 4:**
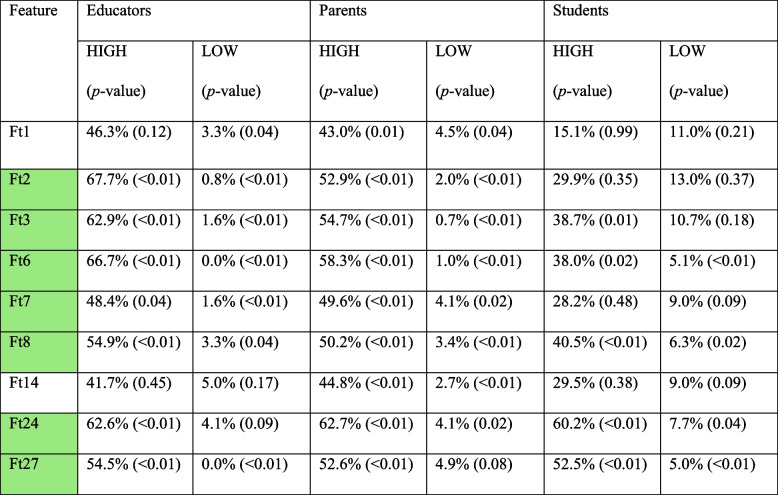
Significant features per Stakeholder

Features that are significant (i.e. p-value HIGH & *p*-value LOW <0.05) to at least one stakeholder group are included. Significant deviations for HIGH & LOW are highlighted, in green the selected features according to methodology and Figure 1 (i.e. S ∪ (E ∩ P)).

### Regional variation

**Fig. 4:**
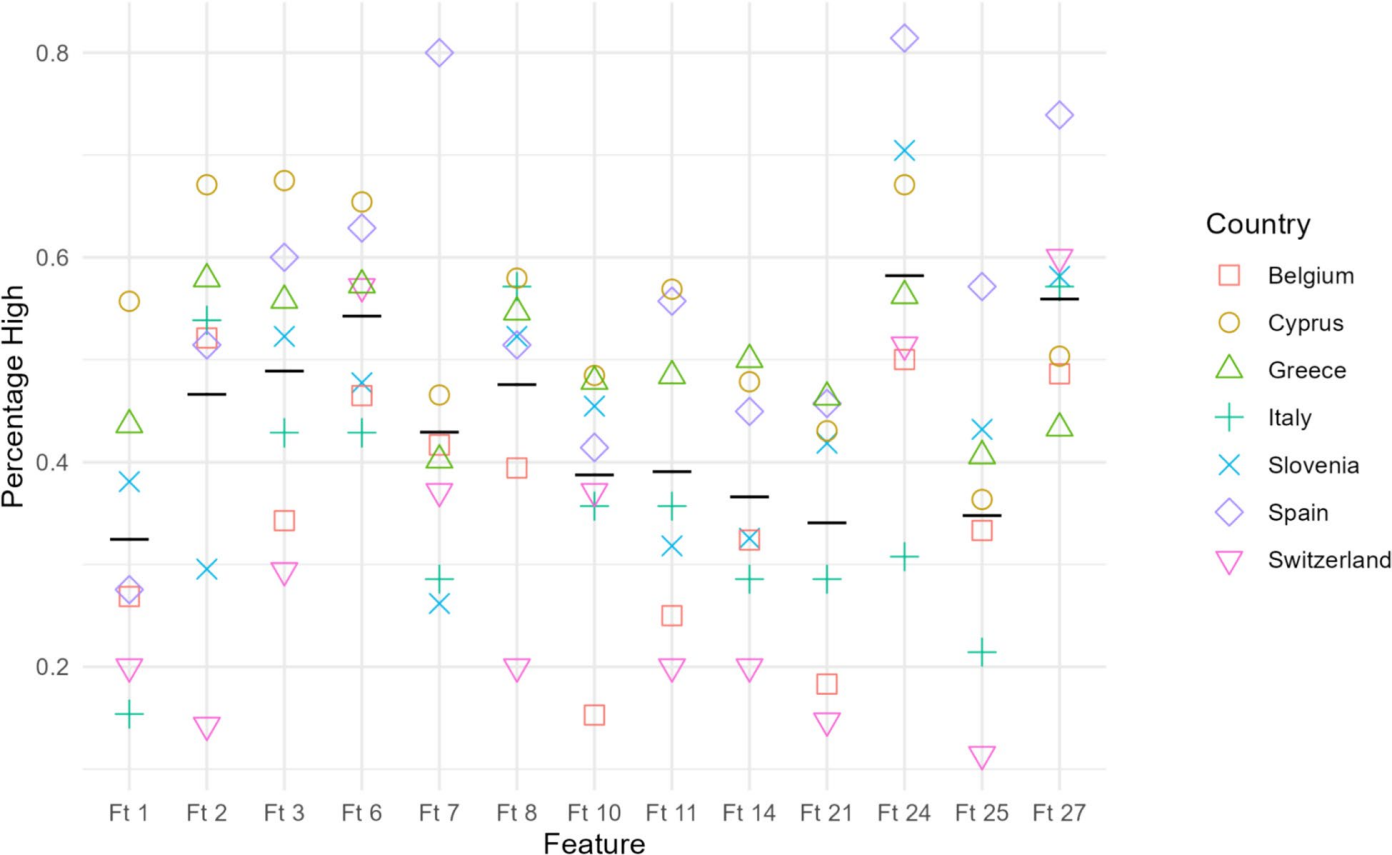
HIGH per Feature and Country

**Fig. 5:**
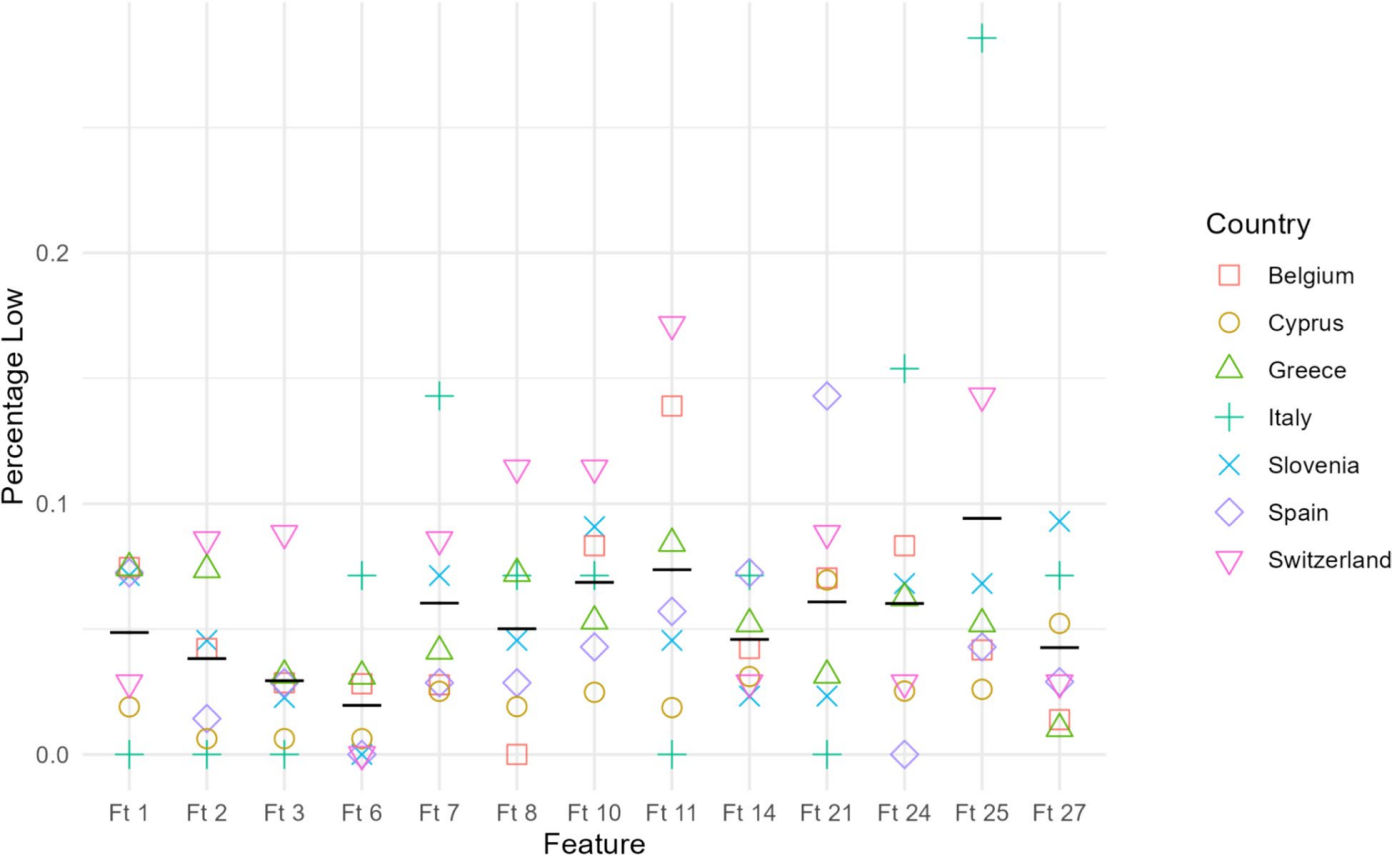
LOW per Feature and Country

Country-specific analysis revealed notable differences in the feature preferences, as illustrated in Figs. 4 and 5. The figures present respectively the HIGH and LOW for each of the thirteen “possibly important” features per country. For every feature, the black bar represents the average value across all countries (i.e., average HIGH in Fig. 4, average LOW in Fig. 5).

Between-country contrasts (Table 5) corroborate the patterns shown in Figs. 4 and 5. After Bonferroni adjustment, the Wilcoxon rank-sum tests identify 14 significant feature-level differences, almost all involving Cyprus, which accounts for nine distinct features. Cyprus participants assign significantly higher importance than Belgium, Italy, or Switzerland to Ft 2, Ft 3, Ft 5, Ft 8, Ft 9, Ft 11, Ft 12, Ft 18, and Ft 25. Spain shows elevated ratings only for Ft 24, where it out-scores Italy. The direction column in Table 5 confirms that no country ever surpasses Cyprus on any feature, underscoring Cyprus’ consistently higher valuations across the significant contrasts.

**Table 5:** Between-country contrasts, Wilcoxon Test*

Feature	Country: A	Country: B	Higher rating	*p*-value
Ft 2	Cyprus	Switzerland	Cyprus	< 0.01
Ft 3	Cyprus	Switzerland	Cyprus	0.01
Ft 5	Belgium	Cyprus	Cyprus	< 0.01
Ft 8	Cyprus	Switzerland	Cyprus	0.02
Ft 9	Spain	Cyprus	Cyprus	0.01
Ft 11	Belgium	Cyprus	Cyprus	< 0.01
Ft 11	Cyprus	Switzerland	Cyprus	< 0.01
Ft 12	Belgium	Cyprus	Cyprus	0.03
Ft 18	Italy	Cyprus	Cyprus	< 0.01
Ft 24	Italy	Spain	Spain	< 0.01
Ft 25	Italy	Cyprus	Cyprus	0.03
Ft 25	Cyprus	Switzerland	Cyprus	0.02

^*^Only feature–country pairs with statistically significant differences are shown. All reported *p*‑values are Bonferroni‑adjusted

These inferential results map directly onto the Figures we provided. In Fig. 4 (percentage of High), the yellow circles representing Cyprus consistently appear at the top of the y-axis for every feature identified in Table 5, while the symbols for Belgium (red squares), Italy (turquoise crosses), and Switzerland (pink triangles) lie noticeably lower. The opposite pattern is seen in Fig. 5 (percentage of Low), where Cyprus clusters near zero and the comparison countries exhibit higher low-importance values.

Spain shows a distinct peak only for Ft 24, where the purple diamond occupies the uppermost point in Fig. 4 and the corresponding lowest point in Fig. 5, matching the Spain > Italy contrast. No such pattern is visible for Ft 25, which aligns with the fact that all Ft 25 contrasts involve Cyprus rather than Spain.

Thus, the direction and magnitude of every statistically significant Wilcoxon contrast in Table 5 are visually anticipated by the country clusters in Figs. 4 and 5, confirming that the formal tests reflect the clear separations seen in the percentage plots.

## Discussion

The results of this study offer valuable insights into stakeholder priorities for developing effective digital health promotion (DHP) tools targeting adolescents across Europe. Our study incorporates responses across seven European countries and three stakeholder groups, including responses from non-binary and mixed migration background individuals (not necessarily representative or proportional amount as it was out of scope). Our findings highlight notable differences in perceived feature importance across stakeholder groups—students, educators, and parents—and significant variations among participating countries. Such disparities suggest that stakeholders hold distinct priorities driven by their roles and contextual backgrounds.

Educators and parents consistently prioritized evidence-based, practical, and economically accessible features. Conversely, adolescent users predominantly emphasized usability features, such as smartphone accessibility, regularly updated content, and free access. These differences underscore the essential balance between educational, parental, and adolescent perspectives in designing effective health interventions. Our methodological emphasis on integrating stakeholder-specific thresholds for feature importance allowed us to identify core features with universal appeal, while also acknowledging subgroup preferences, thereby optimizing both relevance and user engagement.

Country-level analysis highlighted significant variations in feature preferences, particularly between Switzerland and other participating countries. Swiss participants rated several features as less important compared to respondents from other nations, while participants from Spain consistently ranked certain features highly. These country-specific differences underline the importance of culturally sensitive and adaptable DHP tools, responsive to regional health priorities and local stakeholder expectations. Variations in stakeholder representation within countries further influenced preferences. For instance, parental dominance in Cyprus and Belgium may have contributed to prioritizing practical guidance and evidence-based information, whereas the greater educator participation in Greece likely favored structured educational content. Italy's comparatively low overall response rate, particularly among educators, could explain the absence of strong feature preferences within that context.

### A universal backbone emerged

Practical ways to improve behaviors (Ft 6) and Regularly updated content (Ft 8) were the only features rated “very important” and seldom “unimportant” by *all* respondents—students, educators, and parents—irrespective of national context. Neither feature showed a significant between-country shift in Table 5, and both occupy the extreme upper-right quadrant of Figs. 4 and 5. These items, therefore, constitute the non-negotiable core of any future platform.

### Stakeholder-specific layers are equally clear

Evidence-based fact sheets (Ft 2) and Information on multiple health behaviours (Ft 3) and Budget friendly information (Ft 7) were valued by educators and parents but not by students. Retaining these three elements as optional layers—activated only when the corresponding stakeholder opts-in—allows the DHP tools to remain streamlined for students while delivering depth where it is most appreciated.

### Country contrasts point to targeted add-ons

Cyprus displayed the broadest demand, outscoring comparison nations on nine features (Ft2, Ft3, Ft5, Ft8, Ft9, Ft11, Ft12, Ft18, and Ft25). Spain showed a more focused pattern, with a single significant peak on *App delivered for free* (Ft24), where it exceeded Italy. A modular architecture that activates, or suggests, these components only for users in the corresponding country can accommodate local preferences.

Limitations of this study include potential bias due to disproportionate stakeholder representation, especially the overrepresentation of parents, which may affect generalizability. Although subgroup analyses aimed to mitigate this issue, more balanced stakeholder involvement would further enhance the robustness of findings. Additionally, the cross-sectional design limits the ability to draw causal inferences or track changes in stakeholder preferences over time. Employing longitudinal designs in future research could address this limitation by capturing evolving needs and preferences. The relatively low response rates from certain countries also limit the representativeness and suggest a need for enhanced participant recruitment strategies. For example, in Cyprus, the sample was dominated by parents, which may explain the country’s strong preference for features such as trusted content and direct access to health professionals. Belgium showed a similar pattern but with a more balanced representation. In Greece, educators were the largest group, possibly contributing to more institutionally grounded feature evaluations. Spain and Slovenia had balanced stakeholder representation, while Switzerland had moderate and evenly distributed participation, consistent with its lower overall feature ratings. These differences in stakeholder composition likely influenced national-level feature preferences and should be considered when interpreting cross-country variation.

Our survey data serve as a foundation for identifying features that stakeholders consider vital for DHP tools. The cross-sectional nature of this data allows us to explore variations in feature importance across different stakeholder groups and countries, revealing insights into cultural and contextual preferences. By actively involving students, educators, parents, health professionals, and policymakers in both the design and implementation phases, interventions can be tailored to meet educational, cultural, and public health standards, ensuring their relevance and effectiveness. Although current bibliography lacks evidence, specifically, from multi-country, multi-stakeholder studies examining the requirements for digital health tools toward cancer prevention in adolescence, Zarnowiecki and colleagues [18] have identified specific preferences via user-testing research (focusing on parents). Specifically, they report the preference that digital health platforms should provide interactive ‘how to’ components (corresponding to our Ft6), activities parents and children can do together (corresponding to our Ft10), and, information and feedback personalized or tailored to users’ needs (corresponding to our Ft1). These findings partially align with our seven high preference features (green area of Fig. 1), since Ft6 is also selected from our cross-country and cross-stakeholder analysis while Ft1 is only significant for parents (Table 4) and Ft10 is in the list of the 13 possible important features but does not have the required deviation significance to be included. The presented analysis, and the resulting feature prioritization, ultimately aids in the development of culturally adaptable DHP tools that are aligned with the expectations of diverse user groups across Europe.

## Conclusion

This study asked which design elements of a school-based digital intervention are regarded as most important for fostering cancer-preventive habits among European adolescents. By analyzing 28 candidate features across seven countries and three stakeholder groups, we obtained a hierarchy of priorities that can guide both software architecture and implementation strategy.

The methodological approach of comparing high and low importance ratings across stakeholder groups and countries provided a systematic framework for feature identification. This strategy successfully highlighted universally valued core features and group-specific preferences, offering actionable insights for tailored intervention design. Future research should delve deeper into understanding the reasons behind student preferences and identify strategies to make widely valued features more engaging to adolescent users.

Ultimately, this study underscores the critical importance of stakeholder co-creation in developing effective DHP tools. Implementing this structure will accelerate adolescent cancer-prevention efforts and provide a reproducible model for future digital health-promotion initiatives across Europe. Moving forward, targeted engagement strategies that respect demographic and regional preferences will be essential for the successful development and widespread adoption of DHP tools, significantly advancing adolescent cancer prevention and health promotion across Europe.

## Supplementary Information

Supplementary Material 1.

Supplementary Material 2. 

## Data Availability

All data analysed during this study are included in this published article and its supplementary information files.

## Abbreviations

DHP: Digital Health Promotion
IP: Internet Protocol
