# Supplementary material for: Key factors in developing effective digital health promotion tools for cancer prevention and health behavior change in adolescence through a multi-country survey
Source: BMC Public Health. 2026 Feb 21;26:1023. doi: 10.1186/s12889-026-26412-6 (PMC13032310; doi:10.1186/s12889-026-26412-6)
Supplement: Supplementary file 2 — Supplementary Material 2. [file 12889_2026_26412_MOESM2_ESM.pdf]

Supplementary Information File 1: Analysis Code for DHP Tools

2024-06-13

```
Data<-read.xlsx("Supplementary_Data.xlsx")

##########################################################
# 5. MAIN DATA (features) - age is NOT needed here
##########################################################

data <- Data[, -c(1:8, 10:18)]
data <- data[, c(1, ncol(data), 2:(ncol(data) - 1))]
colnames(data)[1] <- "Stakeholder"

# convert "" to NA
data <- data %>%
  mutate(across(everything(), ~ ifelse(.x == "", NA, .x)))

# remove rows with too many missing feature responses
qq  <- is.na(data[, 3:ncol(data)])
qq1 <- apply(qq, 1, sum)
a   <- floor(0.2 * 28)
aa  <- which(qq1 > a)
if (length(aa) > 0) data <- data[-aa, ]

##########################################################
# 6. SIDE DATA (demographics) - WE ADD AGE HERE
##########################################################

data1 <- Data[, -c(1:7, 11:18)]
data1 <- data1[, c(1, 3, 2, ncol(data1), 4:(ncol(data1) - 1))]
colnames(data1)[1:3] <- c(
  "Gender",
  "Were you born in the country you currently live in",
  "Stakeholder"
)

data1 <- data1 %>%
  mutate(across(everything(), ~ ifelse(.x == "", NA, .x)))

# >>> ADD AGE FROM THE ORIGINAL Data <<<
age_col_name <- "Age"   # <<< CHANGE THIS if your age column has a different name
data1 <- data1 %>%
  mutate(Age = as.numeric(Data[[age_col_name]]))

# optional: restrict to realistic ages
data1 <- data1 %>%
  mutate(Age = ifelse(Age < 16 | Age > 100, NA, Age))
```

```r
# apply same missingness filter if you want consistency
qq  <- is.na(data1[, 4:(ncol(data1) - 1)])  # exclude Age from this check
qq1 <- apply(qq, 1, sum)
a   <- floor(0.2 * 28)
aa  <- which(qq1 > a)
if (length(aa) > 0) data1 <- data1[-aa, ]


######A function usefull for later:#####
split_title <- function(title, max_words = 7) {
  words <- unlist(strsplit(title, " "))
  if (length(words) > max_words) {
    split_point <- ceiling(length(words) / 2)
    title <- paste(paste(words[1:split_point], collapse = " "),
                   paste(words[(split_point + 1):length(words)], collapse = " "),
                   sep = "\n")
  }
  return(title)
}
```

```r
feature_cols <- colnames(data)[3:ncol(data)]

result_table <- data.frame(
  Feature   = feature_cols,
  FtCode    = paste0("Ft", seq_along(feature_cols)),
  Count_1   = NA_integer_,
  Count_2   = NA_integer_,
  Count_3   = NA_integer_,
  Count_4   = NA_integer_,
  Count_5   = NA_integer_,
  Total_N   = NA_integer_
)

for (i in seq_along(feature_cols)) {
  feat      <- feature_cols[i]
  responses <- data[[feat]]
  responses <- factor(
    responses,
    levels = c(
      "1: Not important at all",
      "2: Not that important",
      "3: Not sure",
      "4: Important",
      "5: Very important"
    )
  )

  counts <- table(responses, useNA = "no")

  result_table$Count_1[i] <- counts["1: Not important at all"]
  result_table$Count_2[i] <- counts["2: Not that important"]
  result_table$Count_3[i] <- counts["3: Not sure"]
  result_table$Count_4[i] <- counts["4: Important"]
```

```
  result_table$Count_5[i] <- counts["5: Very important"]
  result_table$Total_N[i] <- sum(!is.na(data[[feat]]))
}

Table2 <- result_table[, 2:8]
colnames(Table2) <- c(
  "Feature Code",
  "Rate=1",
  "Rate=2",
  "Rate=3",
  "Rate=4",
  "Rate=5",
  "Total Responses"
)

kable(Table2, caption = "Distribution of Ratings per feature and corresponding LOW
      and HIGH rating proportions (Manuscript Table 2) ")
```

Table 1: Distribution of Ratings per feature and corresponding LOW and HIGH rating proportions (Manuscript Table 2)

Feature Code	Rate=1	Rate=2	Rate=3	Rate=4	Rate=5	Total Responses
Ft1	9	15	54	209	191	478
Ft2	4	13	32	178	259	486
Ft3	3	9	23	186	266	487
Ft4	5	17	69	230	163	484
Ft5	13	38	75	155	208	489
Ft6	3	4	17	182	283	489
Ft7	6	14	52	190	227	489
Ft8	2	17	23	200	246	488
Ft9	5	47	108	219	107	486
Ft10	5	22	51	211	201	490
Ft11	4	29	46	191	220	490
Ft12	19	77	138	175	80	489
Ft13	15	65	107	206	88	481
Ft14	2	19	31	233	204	489
Ft15	10	31	81	228	133	483
Ft16	8	41	67	233	139	488
Ft17	6	39	90	218	135	488
Ft18	8	21	72	249	134	484
Ft19	4	19	34	257	176	490
Ft20	10	39	80	244	109	482
Ft21	3	30	49	219	184	485
Ft22	15	45	131	196	95	482
Ft23	14	84	107	196	83	484
Ft24	5	17	31	129	306	488
Ft25	9	18	61	212	185	485
Ft26	5	41	63	207	167	483
Ft27	5	13	42	164	259	483
Ft28	13	48	56	220	148	485

```
write.csv(Table2,"Table_2.csv")

########################################################
# Demographic plots (Stakeholder, Gender, Age)
########################################################

make_country_stack <- function(df, resp_col,
                               bar_title   = resp_col,
                               palette     = "Set2",
                               legend_lab  = "Responses") {
  resp_sym <- rlang::sym(resp_col)

  plot_dat <- df %>%
    select(Country, Response = !!resp_sym) %>%
    drop_na() %>%
    count(Country, Response, name = "Count")

  bar_tot <- plot_dat %>%
    group_by(Country) %>%
    summarise(Total = sum(Count), .groups = "drop")

  legend_tot <- plot_dat %>%
    group_by(Response) %>%
    summarise(Total = sum(Count), .groups = "drop")

  ggplot(plot_dat, aes(x = Country, y = Count, fill = Response)) +
    geom_col() +
    coord_flip() +
    geom_text(
      aes(label = Count),
      position = position_stack(vjust = 0.5),
      colour = "white", size = 3
    ) +
    geom_text(
      data = bar_tot,
      aes(x = Country, y = Total, label = Total),
      inherit.aes = FALSE,
      nudge_y = 5, hjust = 0, size = 3
    ) +
    scale_fill_brewer(
      palette = palette,
      name    = legend_lab,
      labels  = paste0(legend_tot$Response,
                    " (", legend_tot$Total, ")")
    ) +
    labs(y = "Count", x = NULL, title = bar_title) +
    theme_minimal()
}

p1 <- make_country_stack(
  data1,
  resp_col    = "Stakeholder",
  bar_title   = "Stakeholder distribution by country",
```

```r
  palette    = "Set2",
  legend_lab = "Stakeholder"
)

p2 <- make_country_stack(
  data1,
  resp_col   = "Gender",
  bar_title  = "Gender distribution by country",
  palette    = "Pastel1",
  legend_lab = "Gender"
)

# --- Age: continuous boxplots ----

p_age_country <- ggplot(data1, aes(x = Country, y = Age, colour = Country)) +
  geom_boxplot(outlier.alpha = 0.5, fill = NA) +
  coord_flip() +
  scale_colour_brewer(palette = "Dark2") +
  labs(title = "Age distribution by country",
       x = NULL, y = "Age (years)")

p_age_stakeholder <- ggplot(data1, aes(x = Stakeholder, y = Age, fill = Stakeholder)) +
  geom_boxplot(outlier.alpha = 0.5) +
  coord_flip() +
  scale_fill_brewer(palette = "Set2") +
  labs(
    title = "Age distribution by stakeholder group",
    x = NULL,
    y = "Age (years)"
  )

# Combined demographic figure (2×2)
final_plot <- (p1 / p2 / p_age_stakeholder)
plot(final_plot)
```

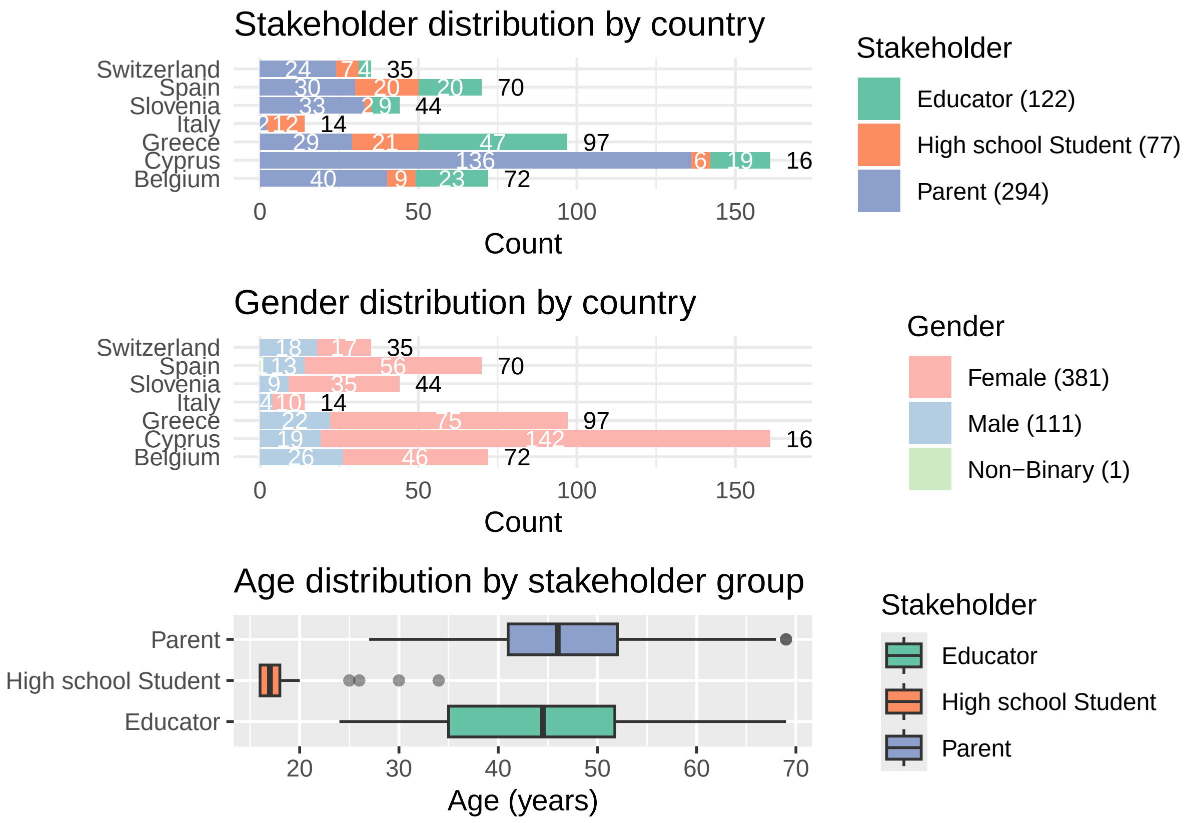


```
ggsave("Demographic_Overview_with_Age.png",
       final_plot,
       width = 10, height = 10, dpi = 300)

########################################################
# 9. Demographic table including age
########################################################

demo_table <- data1 %>%
  group_by(Stakeholder) %>%
  summarise(
    N             = n(),
    Median = median(Age, na.rm = TRUE),
    Mean_Age      = mean(Age, na.rm = TRUE),
    SD_Age        = sd(Age, na.rm = TRUE),
    Min_Age       = min(Age, na.rm = TRUE),
    Max_Age       = max(Age, na.rm = TRUE),
    Male          = sum(Gender == "Male", na.rm = TRUE),
    Female        = sum(Gender == "Female", na.rm = TRUE),
    BornInCountry_Yes =
      sum(`Were you born in the country you currently live in` == "Yes",
          na.rm = TRUE)
  )

#kable(demo_table,
     # caption = " Demographic characteristics by stakeholder group (including age)")
```

```r
perc<-matrix(rep(NA,4*(dim(data)[2]-2)),ncol=4)
for(i in 3:dim(data)[2]){
  tt<-table(data[,i])/dim(data[complete.cases(data[,i]),])[1]
  perc[i-2,3]<-as.numeric(tt[names(tt)=="5: Very important"])
  perc[i-2,4]<-as.numeric(tt[names(tt)=="2: Not that important"]) + as.numeric(tt[names(tt)== "1: Not in
  perc[i-2,1]<-colnames(data)[i]
  perc[i-2,2]<-paste("Ft",i-2)
}

Perc<-data.frame(perc)
Perc[,3:4]<-apply(Perc[,3:4],2,as.numeric)
colnames(Perc)<-c("Orgiginal Question","Code","High","Low")
Perc[,3:4]<-round(Perc[,3:4],4)

######Super Important#####
HIGH<-mean(Perc$High)
LOW<-mean(Perc$Low)




# Print the filtered data
top_left_data <- Perc %>%
  filter(Low < LOW & High > HIGH)


qq<-c(top_left_data[,1])
qq1<-paste("Ft",which(colnames(data) %in% qq)-2)
ImpQue<-data.frame(qq1,qq)
#row.names(ImpQue)<-NULL
#ImpQue
#xtable(ImpQue)


# Now plot it with black and white colors
Perc$cc <- ifelse(Perc[, 1] %in% ImpQue[, 2], "A", "B")

p <- ggplot(Perc, aes(x = Low, y = High, color = cc)) +
  geom_point(size = 3, alpha = 0.8) +  # Add points with color and transparency
  geom_text(aes(label = Code), size = 3, vjust = -0.5) +  # Add text labels
  geom_vline(xintercept = mean(Perc$Low), linetype = "dashed", color = "red") +  # Add vertical line at
  geom_hline(yintercept = mean(Perc$High), linetype = "dashed", color = "red") +  # Add horizontal line
  labs(title = "Figure 3: All countries and stakeholders",
       x = "Percentage of answering Not important at all/Not important",
       y = "Percentage of Very important") +
  scale_color_manual(values = c("A" = "orange", "B" = "black")) +  # Set manual colors
  theme_minimal() +
  theme(
    legend.position = "none",  # Remove the legend
    plot.margin = margin(20, 20, 20, 20)  # Adjust the margin as needed
  )

print(p)
```

Figure 3: All countries and stakeholders

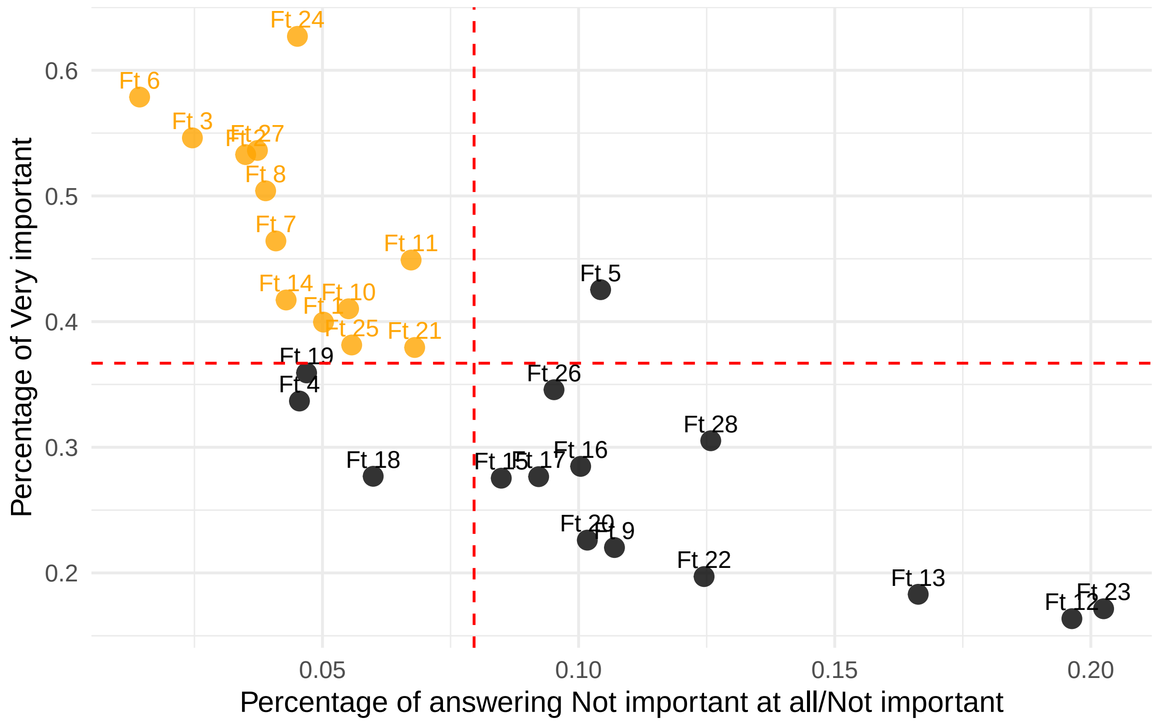


```r
ggsave("MainGraph.PNG")


# Print the plot
##ggsave(filename = "Critical.png", plot = p, width = 8, height = 6, units = "in")


#######TEST##########
n<-dim(data)[1]
mh<-mean(Perc$High)
ml<-mean(Perc$Low)

AllimpFeat1<-Perc[Perc$`Orgiginal Question` %in% ImpQue[,2],-c(2,5)]
AllimpFeat1[,2:3]<-floor(AllimpFeat1[,2:3]*n)
AllimpFeat<-AllimpFeat1


#test clm
#sum((n*AllimpFeat$High) < 5 )#0 so we proceed normaly
#proceed normali
High<-numeric(dim(AllimpFeat)[1])
for(i in 1:dim(AllimpFeat)[1]){High[i]<-round(prop.test(AllimpFeat$High[i],n,mh,alternative = "greater"


#test clm
#sum((n*AllimpFeat$Low) < 5 )#0 so we proceed normaly
```

```
Low<-numeric(dim(AllimpFeat)[1])
for(i in 1:dim(AllimpFeat)[1]){Low[i]<-round(prop.test(AllimpFeat$Low[i],n,ml,alternative = "less", corr

Test<-ifelse(High<0.05 & Low<0.05, "Yes", "No")
Res<-data.frame(ImpQue,High,Low,Test)
#Res[which(Res$Test == "Yes"),1]
colnames(Res)[1]<-"Feauture"
colnames(Res)[2]<-"Question"




library(knitr)
library(kableExtra)

# Make Question wrap-able: replace dots with spaces and collapse multiple spaces
Res$Question <- gsub("\\.+", " ", Res$Question)
Res$Question <- gsub("\\s+", " ", trimws(Res$Question))

kbl(
  Res,
  format   = "latex",
  booktabs = TRUE,
  escape   = TRUE,
  caption  = " p-values of the proportion test of Possibly Important Features. Significant difference fr
  (Manuscript Table 3)."
) %>%
  column_spec(1, width = "1.4cm") %>%   # Feature (keeps it narrow)
  column_spec(2, width = "7.2cm") %>%   # Question (forces wrapping)
  kable_styling(
    font_size = 8,
    latex_options = c("hold_position", "scale_down")
  )
```

```
# 4) Export the correct object to CSV (your previous line exported Table2 by mistake)
#write.csv(Res, "Table_3.csv", row.names = FALSE)
```

```
##############################################################################
#  TABLE 4 : Sub-group testing for significant features
#            - ONE SCRIPT -
##############################################################################


#   labels for High / Low answers
high_label <- "5: Very important"
low_labels <- c("1: Not important at all", "2: Not that important")
data4<-data[,-2]


#   0. SHORT CODES FOR EVERY QUESTION
question_cols  <- setdiff(names(data4), "Stakeholder")            # survey items
feature_codes  <- setNames(paste0("Ft", seq_along(question_cols)),
                           question_cols)                        # Ft1, Ft2, …
```

Table 2: p-values of the proportion test of Possibly Important Features. Significant difference from the mean in both axes (Manuscript Table 3).

Feauture	Question	High	Low	Test
Ft 1	Tailored personalised content incl for age gender low income	0.0661	0.0056	No
Ft 2	Trusted evidence based content and source of information e g health professional such as a dietitian endorsed by a university or government organisation	0.0000	0.0001	Yes
Ft 3	Information on multiple health behaviours e g diet physical activity sedentary BMI	0.0000	0.0000	Yes
Ft 6	Practical ways to improve behaviours how to guidance	0.0000	0.0000	Yes
Ft 7	Budget friendly information i e suggestions that do not have high economic impact	0.0000	0.0007	Yes
Ft 8	Regularly updated content	0.0000	0.0004	Yes
Ft 10	Features relevant for to involve the whole family e g games area or activities for children cooking with children sections for parents	0.0243	0.0208	Yes
Ft 11	Ability to post questions to health professionals e g via a live chat interface contact box video chat or regular contact with health professionals	0.0001	0.1495	No
Ft 14	Recipes budget friendly child friendly quick healthy linked to seasonal produce	0.0121	0.0012	Yes
Ft 21	Resources related to local area e g open sport places farmers markets message board for events	0.2840	0.1495	No
Ft 24	App delivered for freeApp delivered for free	0.0000	0.0021	Yes
Ft 25	In app search function	0.2532	0.0208	No
Ft 27	Accessible via smartphone	0.0000	0.0002	Yes

```
#   1. LONG FORMAT WITH FLAGS
long_dat <- data4 %>%
  pivot_longer(all_of(question_cols),
               names_to  = "Question",
               values_to = "Response") %>%
  filter(!is.na(Response)) %>%
  mutate(
    Code       = feature_codes[Question],
    High_flag = Response == high_label,
    Low_flag  = Response %in% low_labels
  )

#   2. COUNTS & PROPORTIONS PER STAKEHOLDER × FEATURE
summary_tbl <- long_dat %>%
  group_by(Stakeholder, Question, Code) %>%
  summarise(
    n_resp    = n(),
    n_high    = sum(High_flag),
    n_low     = sum(Low_flag),
    perc_high = n_high / n_resp,
    perc_low  = n_low  / n_resp,
    .groups   = "drop"
  )

#   3. STAKEHOLDER-LEVEL REFERENCE MEANS
```

```
means_tbl <- summary_tbl %>%
  group_by(Stakeholder) %>%
  summarise(
    HIGH_mean = mean(perc_high),
    LOW_mean  = mean(perc_low ),
    .groups   = "drop"
  )

summary_tbl <- left_join(summary_tbl, means_tbl, by = "Stakeholder")

#   4. QUADRANT, TWO PROP-TESTS, SigBoth
summary_tbl <- summary_tbl %>%
  rowwise() %>%
  mutate(
    quadrant = ifelse(perc_low < LOW_mean & perc_high > HIGH_mean, "A", "B"),

    pHigh = prop.test(n_high, n_resp,
                      p = HIGH_mean,
                      alternative = "greater", correct = FALSE)$p.value,

    pLow  = prop.test(n_low,  n_resp,
                      p = LOW_mean,
                      alternative = "less",    correct = FALSE)$p.value,

    SigBoth = ifelse(pHigh < 0.05 & pLow < 0.05, "yes", "no")
  ) %>%
  ungroup()

important_features <- summary_tbl         # keep full results if you need them later

impfeaaat<-important_features[,c("Code","Stakeholder","perc_high","perc_low","pHigh","pLow")]
write.csv(impfeaaat,file="Table4.csv")

#unique(impfeaaat[impfeaaat$pHigh<=0.05 & impfeaaat$pLow<=0.05,"Code"])


#   5. LOGIC THAT DEFINES THE FEATURE SUBSET (YOUR Sig1 … Sig4 STEPS)
Sig1        <- filter(important_features, SigBoth == "yes")
SigStudents <- filter(Sig1, Stakeholder == "High school Student")
SigParent   <- filter(Sig1, Stakeholder == "Parent")
SigEducator <- filter(Sig1, Stakeholder == "Educator")

TotalSigPerStake<-c(union(intersect(SigParent$Code,SigEducator$Code),SigStudents$Code))
StakeImpFeat<-unique(c(SigParent$Code,SigEducator$Code,SigStudents$Code))
FinalTable1<-important_features[which(important_features$Code %in% StakeImpFeat),c("Code","Stakeholder",

FinalTable1[,c("perc_high","perc_low")]<- FinalTable1[,c("perc_high","perc_low")]*100


trunc_dec <- function(x, n) {
  trunc(x * 10^n) / 10^n
}
```

```r
FinalTable<- FinalTable1 %>%
  mutate(
    perc_high_trunc = trunc_dec(perc_high, 2),     # one decimal
    perc_low_trunc  = trunc_dec (perc_low,  3),
    pHigh_trunc     = trunc_dec(pHigh, 2),             # three decimals
    pLow_trunc      = trunc_dec(pLow,  2),

      HIGH = sprintf("%.1f%% (%.3f)", perc_high_trunc, pHigh_trunc),
    LOW  = sprintf("%.1f%% (%.3f)", perc_low_trunc,  pLow_trunc)
  ) %>%
  # …then pivot & kable as before…
  select(Stakeholder, Code, HIGH, LOW)



# 2) Reshape to wide: one row per feature, separate HIGH/LOW for each stakeholder
tbl_wide <- FinalTable %>%
  pivot_wider(
    names_from  = Stakeholder,
    values_from = c(HIGH, LOW),
    names_sep   = "_"
  ) %>%
  # reorder features numerically
  mutate(
    feat_num = parse_number(Code)
  ) %>%
  arrange(feat_num) %>%
  select(-feat_num)

FinalTable
```

```
## # A tibble: 24 x 4
##    Stakeholder         Code  HIGH          LOW
##    <chr>               <chr> <chr>         <chr>
##  1 Educator            Ft27  55.0% (0.000) 0.0% (0.000)
##  2 Educator            Ft24  62.3% (0.000) 4.1% (0.090)
##  3 Educator            Ft7   49.2% (0.030) 1.6% (0.000)
##  4 Educator            Ft3   63.1% (0.000) 1.6% (0.000)
##  5 Educator            Ft6   66.9% (0.000) 0.0% (0.000)
##  6 Educator            Ft14  41.5% (0.460) 5.1% (0.180)
##  7 Educator            Ft8   55.8% (0.000) 3.3% (0.040)
##  8 Educator            Ft2   67.2% (0.000) 0.8% (0.000)
##  9 High school Student Ft27  54.5% (0.000) 5.2% (0.010)
## 10 High school Student Ft24  61.3% (0.000) 8.0% (0.060)
## # i 14 more rows
```

```r
write.csv(FinalTable,"Table4.csv")
kable(FinalTable,caption ="Significant features per Stakeholder. Features that are significant (i.e. p-v
```

Table 3: Significant features per Stakeholder. Features that are significant (i.e. p-value HIGH & p-value LOW <0.05) to at least one stakeholder group are included. Significant deviations for HIGH & LOW are highlighted, in green the selected features according to methodology and Figure 1 (i.e. S (E P)). (Talbe 4 manuscript)

Stakeholder	Code	HIGH	LOW
Educator	Ft27	55.0% (0.000)	0.0% (0.000)
Educator	Ft24	62.3% (0.000)	4.1% (0.090)
Educator	Ft7	49.2% (0.030)	1.6% (0.000)
Educator	Ft3	63.1% (0.000)	1.6% (0.000)
Educator	Ft6	66.9% (0.000)	0.0% (0.000)
Educator	Ft14	41.5% (0.460)	5.1% (0.180)
Educator	Ft8	55.8% (0.000)	3.3% (0.040)
Educator	Ft2	67.2% (0.000)	0.8% (0.000)
High school Student	Ft27	54.5% (0.000)	5.2% (0.010)
High school Student	Ft24	61.3% (0.000)	8.0% (0.060)
High school Student	Ft7	28.9% (0.460)	7.9% (0.060)
High school Student	Ft3	38.4% (0.030)	11.0% (0.230)
High school Student	Ft6	39.5% (0.010)	5.3% (0.010)
High school Student	Ft14	28.6% (0.490)	9.1% (0.100)
High school Student	Ft8	41.5% (0.000)	6.5% (0.020)
High school Student	Ft2	29.7% (0.400)	13.5% (0.450)
Parent	Ft27	52.8% (0.000)	4.9% (0.100)
Parent	Ft24	63.2% (0.000)	3.8% (0.020)
Parent	Ft7	49.8% (0.000)	4.1% (0.030)
Parent	Ft3	55.1% (0.000)	0.7% (0.000)
Parent	Ft6	58.9% (0.000)	1.0% (0.000)
Parent	Ft14	45.2% (0.000)	2.7% (0.000)
Parent	Ft8	50.5% (0.000)	3.4% (0.010)
Parent	Ft2	53.4% (0.000)	2.1% (0.000)

```
qq<-ImpQue[,2]

perc<-matrix(rep(NA,5*length(qq)),ncol=5)
dd<-data[which(data$Country==unique(data$Country)[1]),]
  for(i in 1:length(qq)){

  t1<-which(colnames(dd)==qq[i])
  perc[i,3]<-length(which(dd[,t1]=="5: Very important"))/dim(dd[complete.cases(dd[,t1]),])[1]
  perc[i,4]<- length(which(dd[,t1]=="2: Not that important" | dd[,t1]=="1: Not important at all" )) /dim

  perc[i,1]<-colnames(data)[which(colnames(data)==qq[i])]
  perc[i,2]<-paste("Ft",which(colnames(data)==qq[i])-2)
  perc[i,5]<-unique(dd$Country)[1]
}
PerC<-perc

perc<-matrix(rep(NA,5*length(qq)),ncol=5)
for(j in 2:7){
  dd<-data[which(data$Country==unique(data$Country)[j]),]
  for(i in 1:length(qq)){
```

```r
    t1<-which(colnames(dd)==qq[i])
  perc[i,3]<-length(which(dd[,t1]=="5: Very important"))/dim(dd[complete.cases(dd[,t1]),])[1]
  perc[i,4]<- length(which(dd[,t1]=="2: Not that important" | dd[,t1]=="1: Not important at all" )) /dim

  perc[i,1]<-colnames(data)[which(colnames(data)==qq[i])]
  perc[i,2]<- paste("Ft",which(colnames(data)==qq[i])-2)

  perc[i,5]<-paste(unique(data$Country)[j])
  }
  PerC<-rbind(PerC,perc)
}

PerC<-data.frame(PerC)
PerC[,3:4]<-apply(PerC[,3:4],2,as.numeric)
PerC[,3:4]<-round(PerC[,3:4],4)
colnames(PerC)<-c("Feature","Code","High","Low","Country")
PerC$Code <- factor(PerC$Code, levels = unique(PerC$Code))

#Prepare for the diagram
mean_df <- PerC %>%
  group_by(Code) %>%
  summarise(
    meanHigh = mean(High, na.rm = TRUE),
    meanLow  = mean(Low,  na.rm = TRUE),
    .groups  = "drop"
  )
custom_shapes <- c(0, 1, 2, 3, 4, 5, 6, 7, 8)  # Use more shapes as needed


#         2. HIGH plot with country points + mean line
p1 <- ggplot(PerC, aes(x = Code, y = High, colour = Country, shape = Country)) +
  geom_point(size = 3, alpha = 0.9) +
  ## mean line per feature
  geom_crossbar(
    data   = mean_df,
    aes(x = Code, y = meanHigh, ymin = meanHigh, ymax = meanHigh),
    inherit.aes = FALSE,
    width  = .5,          # how wide the bar spans in x
    colour = "black",
    fatten = 0            # keeps thickness constant
  ) +
  labs(title ="Figure 4", x = "Feature", y = "Percentage High", colour = "Country", shape = "Country") +
  scale_shape_manual(values = custom_shapes) +
  theme_minimal() +
  theme(legend.position = "right",
        legend.box.margin = margin(0, 0, 0, 20))

#         3. LOW plot with country points + mean line
p2 <- ggplot(PerC, aes(x = Code, y = Low, colour = Country, shape = Country)) +
  geom_point(size = 3, alpha = 0.9) +
  geom_crossbar(
    data   = mean_df,
    aes(x = Code, y = meanLow, ymin = meanLow, ymax = meanLow),
```

```
    inherit.aes = FALSE,
    width  = .5,
    colour = "black",
    fatten = 0
  ) +
  labs(title="Figure 5",x = "Feature", y = "Percentage Low", colour = "Country", shape = "Country") +
  scale_shape_manual(values = custom_shapes) +
  theme_minimal() +
  theme(legend.position = "right",
        legend.box.margin = margin(0, 0, 0, 20))

#          4. Display
print(p1)
```

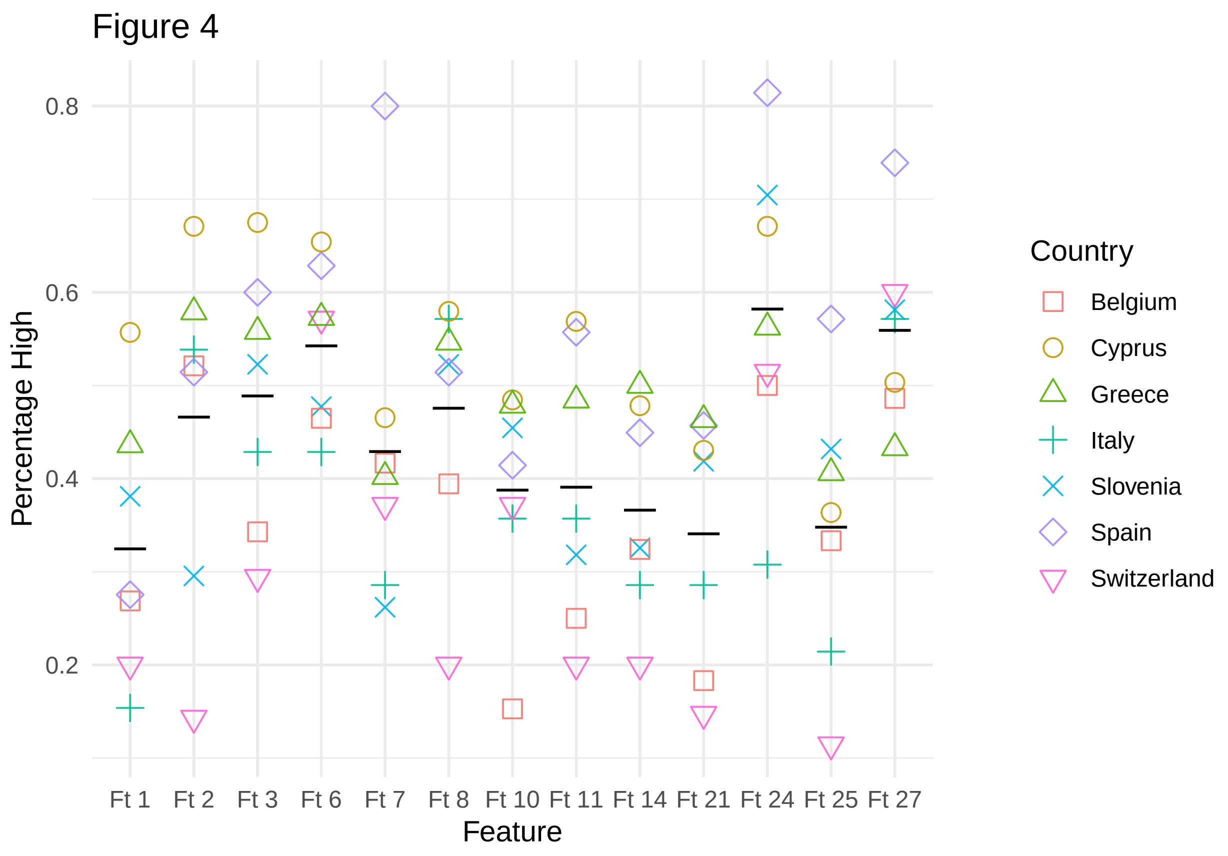


```
ggsave("CountriesHigh.PNG")
print(p2)
```

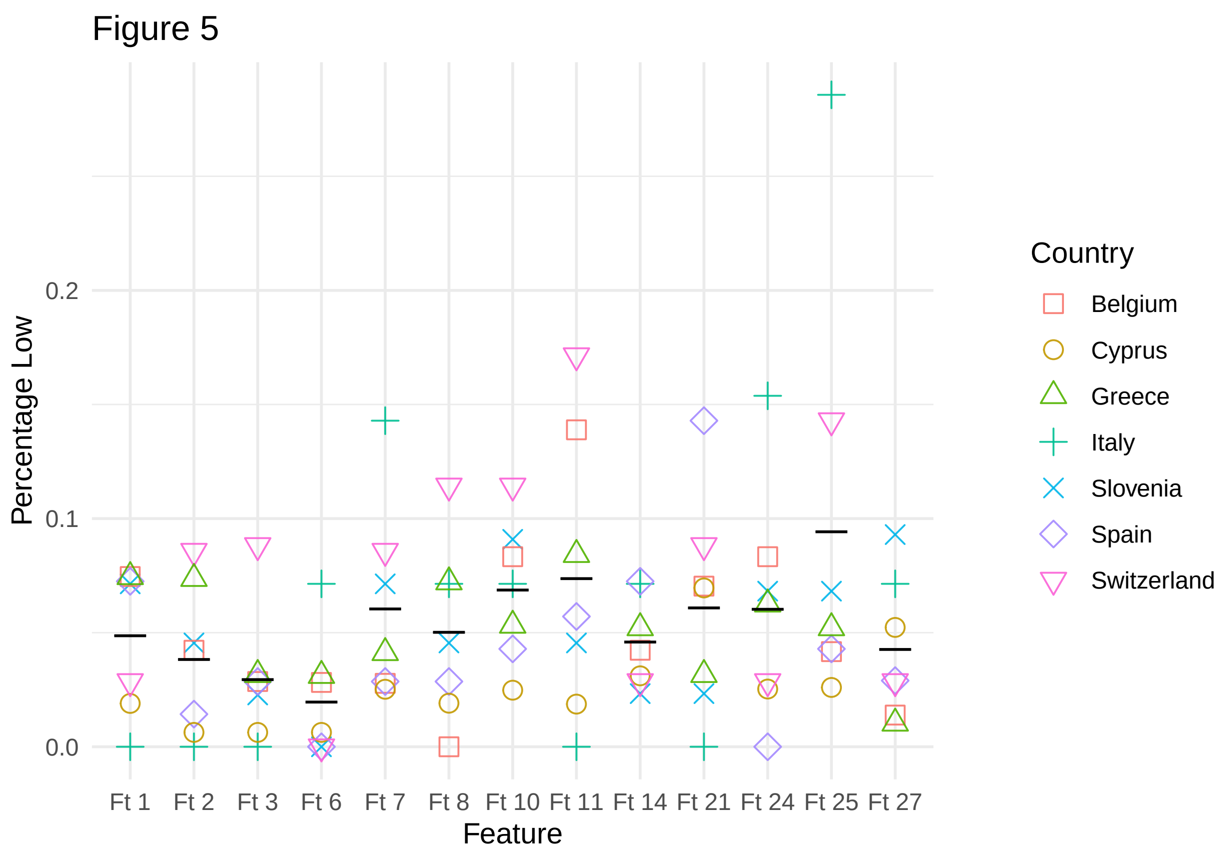


```
ggsave("CountriesLow.PNG")








#####Testing#####


library(dplyr)
library(tidyr)



df<-data[,-1]

valid_responses <- c("5: Very important", "2: Not that important", "1: Not important at all")
feature_cols <- colnames(data)[3:30]
countries <- unique(data$Country)


df[feature_cols] <- lapply(df[feature_cols], function(col) {
  ifelse(col %in% valid_responses, col, NA)
```

```
})



group1<- df[df$Country== countries[1],-1]
group2<-df[df$Country== countries[2],-1]



recode_vals <- function(x) {
  case_when(
    x == "5: Very important" ~ 5,
    x == "2: Not that important" ~ 2,
    x == "1: Not important at all" ~ 1,
    TRUE ~ NA_real_
  )
}
df[feature_cols] <- lapply(df[feature_cols], recode_vals)

# Step 2: Wilcoxon test for each feature and country pair
countries <- unique(df$Country)
country_pairs <- combn(countries, 2, simplify = FALSE)

results <- data.frame()

for (feat in feature_cols) {
  for (pair in country_pairs) {

    c1 <- pair[1];  c2 <- pair[2]

    g1 <- df %>% filter(Country == c1) %>% pull(feat) %>% na.omit()
    g2 <- df %>% filter(Country == c2) %>% pull(feat) %>% na.omit()

    if (length(g1) >= 2 & length(g2) >= 2) {

      ## -- run the test safely --
      test <- tryCatch(
        wilcox.test(g1, g2, exact = FALSE, conf.int = TRUE),
        warning = function(w) suppressWarnings(wilcox.test(g1, g2, exact = FALSE, conf.int = FALSE)),
        error   = function(e) wilcox.test(g1, g2, exact = FALSE, conf.int = FALSE)
      )

      ## Hodges-Lehmann shift (may be NA)
      hl <- if (!is.null(test$estimate)) as.numeric(test$estimate) else median(g1) - median(g2)

      ## direction string
      direction <- case_when(
        hl  >  0    ~ paste(c1, "higher"),
        hl  <  0    ~ paste(c2, "higher"),
        TRUE        ~ "tie"
      )

      results <- rbind(results, data.frame(
```

```
        Code         = feature_codes[feat],    # Ft1, Ft2, …
        Country1     = c1,
        Country2     = c2,
        Direction    = direction,
        HL.shift     = round(hl, 3),
        p.value      = test$p.value,
        W.statistic  = test$statistic,
        N_C1         = length(g1),
        N_C2         = length(g2)
      ))
    }
  }
}

results$p.adj <- p.adjust(results$p.value, method = "bonferroni")
sig_results   <- results %>% filter(p.adj < 0.05)

Table5<-sig_results[,c(colnames(sig_results)[1:4],"p.adj")]
row.names(Table5)<-NULL
kable(Table5,caption ="Between-country contrasts, Wilcoxon Test* (manuscript Table 5) ")# %>%   kable_s
```

Table 4: Between-country contrasts, Wilcoxon Test* (manuscript Table 5)

Code	Country1	Country2	Direction	p.adj
Ft2	Cyprus	Switzerland	Cyprus higher	0.0000382
Ft3	Cyprus	Switzerland	Cyprus higher	0.0141027
Ft5	Belgium	Cyprus	Cyprus higher	0.0003347
Ft8	Cyprus	Switzerland	Cyprus higher	0.0202162
Ft9	Spain	Cyprus	Cyprus higher	0.0178426
Ft11	Belgium	Cyprus	Cyprus higher	0.0005602
Ft11	Cyprus	Switzerland	Cyprus higher	0.0001188
Ft12	Belgium	Cyprus	Cyprus higher	0.0312154
Ft18	Italy	Cyprus	Cyprus higher	0.0003380
Ft24	Italy	Spain	Spain higher	0.0077714
Ft25	Italy	Cyprus	Cyprus higher	0.0389980
Ft25	Cyprus	Switzerland	Cyprus higher	0.0295526

```
library(officer)
library(flextable)

# Create a new Word document
doc <- read_docx()

# Add a title (optional)
doc <- doc %>%
  body_add_par("Significant Wilcoxon Test Results -Table5", style = "heading 1") %>%
  body_add_par("")  # empty line

# Add the table
doc <- doc %>%
  body_add_flextable(flextable(sig_results[,c(1:4,8)]))
```

```
# Save the Word file
#print(doc, target = "significant_results.docx")
```
